# 

**Published:** 1896-04

**Authors:** 


### PERSONAL.

Veterinarians Pearson and Hoskins, of Philadelphia, appeared before a committee of the Massachusetts Legislature, at Boston, charged with hearing those who wished to speak relative to the bills now pending before that body looking to a reduction of the Cattle Commission or the placing of this work in the hands of local boards of health. Their voices were raised in appeal not to stay the work of this intelligent and conscientious Commission, but to strengthen it with increased power and privileges.

Prof. Roscoe R. Bell, the new associate-editor, with Prof. Liautard, on the *Review*, while not so well known among Association circles as the latter, will no doubt soon be found in the active pathway of work of our national organization, the Empire State's strong State body, and no better person could be charged with the resuscitation of that once influential and successful organization, the Long Island Veterinary Society.

Professor James L. Robertson, who has recently been seriously ill, is again back to his post of duty at the American Veterinary College, where he has for a long period of years filled so acceptably the chair of theory and practice.

Dr. W. L. Zuill, of Philadelphia, and several members of his family have recently been stricken with diphtheria.

Dr. Nelson P. Hinkley, of Buffalo, and Dr. W. L. Williams, of Bozeman, Montana, have recently been battling with attacks of the grippe.

The Buffalo master-shoers will have in their school of anatomy and horseshoeing the services of veterinarians Willganz and William Somerville.

Dr. George H. Berns, of Brooklyn, has been added to the staff of collaborateurs of the *American Veterinary Review*.

Dr. James A. Waugh, of Allegheny, is out in a recent issue of the *Humane Advocate*, of Pittsburg, with an article on "Docking of Tails of Horses and Cropping of Ears of Dogs."

The February number of the *Farm and Home*, published at Melbourne, Australia, contains a photo-engraving of W. Tyson Kendall, M.R.C.V.S., Principal of the Melbourne Veterinary College.

F. B. McCall, D.V.M., has been added to the staff of instructors connected with the Veterinary Department at Ames, as lecturer on lameness and principles of shoeing.

Dr. W. J. Hinman, of Winnipeg, Manitoba, has had added to his duties, for his city, those of meat- and food-inspector.

Dr. W. V. Lusk, of Ohio, and one of the former members of the State Board of Veterinary Medical Examiners, has accepted a post in the army service as veterinarian to the 2d Cavalry regiment of the United States Army.

Dr. George H. Bailey holds the place of veterinary editor to the *Turf, Farm, and Home*, published at Waterville, Maine.

Veterinarian Schmitt, of Dodgeville, Wisconsin, is just recovering from a painful and serious injury which has confined him to his bed for some weeks.

Veterinarian Johnson, of Moore's Station, Pennsylvania, recently met with a severe mishap, through his horse running away and throwing Mrs. Johnson and the Doctor forcibly from the carriage, injuring both, the former quite seriously.

Dr. Rush S. Huidekoper was one of the judges at the recent National Cat-show in New York City.

The *Allegheny* (Pa.) *Herald* of March 6 has a half-column of news headed "A Sensible Idea," referring to the recent appointment of Dr. J. Stewart Lacock as City Veterinarian. The city, with its $30,000 worth of live-stock, already appreciates the value of expert advice in the selection of horses for the special services required of them and heartily approves of the new innovation.

Dr. William Dougherty, of Baltimore, in the March number of the *American Veterinary Review*, suggests a good arrangement for a frost- and ice-shoe: That the toe-calking be made with three points and the heel-calkings with two points. As these become dulled from use they can be sharpened with a file without removing from the feet.

Drs. W. L. Zuill, Leonard Pearson, and W. Horace Hoskins have been selected by Mayor Warwick, of Philadelphia, as members of a special Board of Examiners to examine applicants for the vacancy in the position of Consulting Veterinarian to the Department of Public Safety.

Dr. W. H. Dalrymple, recently of Atlanta, Georgia, has returned to Baton Rouge, Louisiana, the seat of his former field of labor.

Dr. A. W. Swedberg, recently of Richmond, Virginia, has been appointed an assistant-inspector in the Bureau of Animal Industry, and assigned to duty at St. Louis, Missouri.

Dr. S. S. Field, Jr., of New York City, is touring the country with the play entitled "The Sporting Duchess." He has the general management and direction of the unusual number of horses which make up a very attractive feature of the show.

The health of Dr. William Dougherty, of Baltimore, has been seriously impaired the past winter.

The following well-known veterinarians will officiate in the Boston Horse-show in April next: Drs. Osgood, Howard, Peters, Labaw, and Blackwood.

Dr. C. Barnwell Robinson has been appointed veterinarian to the Health Board of the District of Columbia.

Veterinarian W. T. S. Werntz is a candidate for representative honors in his district, part of West Philadelphia.

Dr. J. Stewart Lacock, of Allegheny City, Pennsylvania, has been appointed city meat- and milk-inspector at a salary of one thousand dollars per year.

Veterinarian W. L. Burt, of Providence, Rhode Island, was a recent visitor to the Buffalo horse-markets, selecting coach-horses for his clients.

Prof. R. S. Huidekoper's bull-terrier, "Cardona," won the cup given by the National Greyhound Club for the best dog of the bull-terrier class at the recent New York Kennel-Club Show.

Veterinarian George H. Bailey, of Maine, says in the Boston *Herald* of March 16 that he does not believe the trotting-horse carrying one hundred and fifty pounds on a level track will ever reach a two-minute gait.

The many friends of Dr. C. P. Lyman will be glad to know that he is again back in the harness, improved in health and bearing evidence of his old-time vigor.

Dr. W. F. Lavery, of Chillicothe, Ohio, who has been called to a position at the Veterinary Department of the Ohio State University at Columbus, Ohio, is a graduate of the Veterinary Department of that institution, class of 1890, and was a former assistant of Prof. H. J. Detmers.

Dr. Alexander Glass has been selected as veterinarian for the Philadelphia Dog-show, which will be held at Industrial Hall on the 14th, 15th, 16th, and 17th of April.

